# A Systematic Review of Cutting-Edge Radar Technologies: Applications for Unmanned Ground Vehicles (UGVs)

**DOI:** 10.3390/s24237807

**Published:** 2024-12-06

**Authors:** Can Ersü, Eduard Petlenkov, Karl Janson

**Affiliations:** Department of Computer and Systems Engineering, Tallinn University of Technology, Ehitajate tee 5, 19086 Tallinn, Estonia; eduard.petlenkov@taltech.ee (E.P.); karl.janson@taltech.ee (K.J.)

**Keywords:** radar, UGV, offroad, machine learning, navigation, object detection

## Abstract

This systematic review evaluates the integration of advanced radar technologies into unmanned ground vehicles (UGVs), focusing on their role in enhancing autonomy in defense, transportation, and exploration. A comprehensive search across IEEE Xplore, Google Scholar, arXiv, and Scopus identified relevant studies from 2007 to 2024. The studies were screened, and 54 were selected for full analysis based on inclusion criteria. The review details advancements in radar perception, machine learning integration, and sensor fusion while also discussing the challenges of radar deployment in complex environments. The findings reveal both the potential and limitations of radar technology in UGVs, particularly in adverse weather and unstructured terrains. The implications for practice, policy, and future research are outlined.

## 1. Introduction

Unmanned ground vehicles (UGVs) are revolutionizing operations in various domains, including precision agriculture, disaster response, industrial automation, and surveillance. These vehicles rely on advanced sensing technologies to achieve autonomy, enabling them to navigate, detect obstacles, and execute complex tasks in real time. While military reconnaissance and defense applications have been significant drivers of UGV development, this work focuses primarily on civilian and industrial applications, addressing the challenges posed by unstructured environments. Such environments—characterized by uneven terrains, dense vegetation, and adverse weather conditions—pose significant obstacles for existing sensors like light detection and ranging (LiDAR) and optical cameras, which are often hindered due to occlusions, clutter, and low visibility. Radar technologies, with their unique ability to penetrate such barriers and perform under extreme conditions, are increasingly pivotal in overcoming these limitations [1].

Radar technology has emerged as a key solution, offering robustness in adverse conditions and long-range obstacle detection. Unlike LiDAR, which excels in high-resolution mapping but is limited in fog, rain, or dusty settings, radar maintains reliable performance in such scenarios [2]. Recent advancements, such as radar-rased simultaneous localization and mapping (SLAM) systems and sensor fusion techniques, further enhance UGVs’ ability to operate effectively in diverse, dynamic, and uncertain conditions [3,4].

The demand for radar technology in UGV applications within unstructured environments is underscored by numerous studies and use cases. For instance, radar’s ability to penetrate environmental occlusions, such as vegetation or dust [5], has been demonstrated to be critical in forestry and agricultural contexts [4,6]. Recent research highlights radar’s efficacy in detecting obstacles hidden by vegetation, which is essential for UGVs deployed in forestry, search-and-rescue missions, and defense operations. In agricultural contexts, radar has been used to navigate uneven terrains and detect objects amid dust or low-visibility conditions, proving its adaptability to challenging field environments. As the scope of UGV applications continues to grow, particularly in areas that require operation in remote or harsh environments, the demand for reliable sensing technologies like radar has increased, solidifying its role as a foundational technology for these applications.

### 1.1. Radar vs. LiDAR: Key Differences

- Environmental robustness: LiDAR excels in generating high-resolution 3D maps but struggles in adverse conditions like heavy rain or fog. Radar, in contrast, reliably penetrates occlusions such as vegetation and dust, making it better suited to off-road environments [7,8].
- Detection range and resolution: Radar systems are capable of detecting objects at long distances with consistent accuracy, a critical feature for path planning in vast outdoor settings. LiDAR, while offering a higher spatial resolution, is constrained due to shorter detection ranges, limiting its applicability in open or expansive terrains [9].
- Weather resilience: Adverse weather conditions like snow, rain, and dusty environments heavily impact LiDAR’s reliability, reducing its effectiveness in obstacle detection. Radar’s insensitivity to these factors enables consistent performance, ensuring reliable UGV operation in diverse environmental conditions [10].
- Cost and energy efficiency: Radar systems are typically more affordable and durable, making them well-suited to deployment in harsh and expansive terrains. In contrast, LiDAR offers greater precision, which makes it the preferred choice for urban or structured environments where detailed mapping is essential [11].

### 1.2. Radar vs. LiDAR: UGV Applications

The choice of radar or LiDAR in UGVs is influenced by the specific requirements of the operational environment. Radar is particularly suitable for the following.

- Off-road navigation: Radar’s ability to penetrate foliage and withstand environmental occlusions makes it ideal for forestry, agriculture, and search-and-rescue operations [12,13].
- Long-range obstacle detection: The superior range of radar allows for early obstacle identification, enhancing safety and operational planning in defense and exploration missions [9].
- Weather-resilient mapping: In terrains prone to rain or fog, radar ensures uninterrupted performance, supporting reliable SLAM and obstacle detection [10].

LiDAR remains indispensable for the following.

- High-precision mapping: Urban environments requiring detailed spatial resolution benefit from LiDAR’s ability to create accurate 3D maps.
- Indoor navigation: LiDAR’s ability to provide precise measurements in structured indoor environments makes it suitable for industrial or warehouse automation tasks [14].

While both radar and LiDAR play pivotal roles in enhancing UGV autonomy, their unique strengths cater to different application domains. For operations in unstructured and adverse environments, radar offers unmatched reliability, while LiDAR provides precision in controlled settings. The integration of these technologies, such as radar–LiDAR fusion, has proven to be an effective approach to leveraging their complementary advantages.

Despite these advantages, integrating radar into UGV systems presents significant technical challenges that have not been fully addressed in the literature. While radar excels in long-range sensing, its resolution in comparison to LiDAR and optical sensors can be limited, making object classification and fine-grained navigation difficult. Furthermore, the fusion of radar data with other sensor modalities, such as LiDAR or cameras, introduces complexity in terms of data synchronization, calibration, and computational overhead [15]. The effective use of radar for SLAM in UGVs also remains a research frontier, particularly in off-road and rural settings where radar data can be sparse or cluttered due to natural obstacles. Recent advancements, such as integrating radar with inertial measurement units (IMUs) and applying robust graph-based algorithms, have shown promise in enhancing radar-SLAM capabilities in such environments [16,17].

### 1.3. Research Questions

This systematic review aims to bridge these knowledge gaps by comprehensively analyzing the current state of radar technologies in UGVs. Specifically, it addresses the following research questions.

- **RQ1:** What are the recent advancements in radar technologies, and how do these innovations enhance the capabilities of UGVs?
- **RQ2:** How do current radar technologies integrate with machine learning and sensor fusion techniques to improve the accuracy and reliability of object detection, navigation, and object tracking in UGVs?
- **RQ3:** What are the emerging trends and future directions in radar technology research for enhancing the capabilities of UGVs?

### 1.4. Organization of the Paper

The paper is structured as follows: Section 2 reviews the existing literature and recent developments in radar technologies for UGVs. Section 3 discusses the integration of radar with other sensors and machine learning techniques. Section 4 highlights key challenges and research gaps in radar applications for UGVs. Section 5 concludes with recommendations and future research directions.

By systematically evaluating advancements and challenges in radar technologies, this review provides a foundation for future research aimed at overcoming the limitations of current UGV systems and paving the way for more robust, autonomous operations across diverse sectors.

## 2. Methods

### 2.1. Eligibility Criteria

This review included studies that focused on the integration of radar technologies in UGVs. Eligible studies were those published in peer-reviewed journals, conference proceedings, and technical reports, focusing on advancements, challenges, and innovations in radar technologies for UGVs. The inclusion criteria required studies to involve applications of radar technology in UGVs, with a particular focus on sensor integration, navigation, object detection, and machine learning. Only studies published in English were included in the review to ensure consistency in language. Boolean operators were used in the search process to broaden the scope and capture a wider range of relevant studies, with the inclusion period set from January 2007 to September 2024, were considered. Excluded were studies that did not explicitly address radar technologies or their integration into UGV systems.

### 2.2. Search Strategy

To enhance transparency, the search strategy is elaborated upon with more specific details.

- Databases: A comprehensive search was conducted across key academic databases, including IEEE Xplore, Google Scholar, arXiv, and the Scopus digital collection using carefully crafted search strings. The strings were designed to capture relevant literature on radar technologies and their integration with UGVs.
- Keywords: The search terms included “radar technologies”, “Unmanned Ground Vehicles”, “autonomous systems”, “sensor integration”, and their variations. Boolean operators and truncation were employed strategically to broaden or narrow search results as needed.
- Inclusion criteria: The inclusion criteria specified a publication period of the last 18 years to ensure currency. Peer-reviewed journal articles, conference proceedings, and pertinent book chapters were included.
- Exclusion criteria: Exclusion criteria were explicitly defined, excluding non-English publications, duplicates, and studies falling outside the scope of radar technologies applied to UGVs.

### 2.3. Selection Process

The selection of studies was performed in two stages. Initially, the titles and abstracts of all retrieved studies were screened by the primary researcher to assess their relevance based on the predefined eligibility criteria. Studies that appeared relevant underwent a full-text review. During this stage, the primary researcher independently evaluated the studies against the eligibility criteria. The work was inspected and reviewed by two supervisors who provided oversight and guidance throughout the process. Any uncertainties or potential disagreements in the selection process were discussed with the supervisors to ensure accuracy and consistency in the final selection of studies.

### 2.4. Data Collection Process

Data extraction was carried out by the primary researcher using a standardized form that was piloted on a subset of studies to ensure consistency and completeness. The primary researcher independently extracted key information from the included studies, including study characteristics (such as authors, year of publication, and country), UGV and radar technology details (such as types of radar used and integration methods), and outcomes (such as advancements, challenges, and innovations).

### 2.5. Data Items

The specific data items extracted from each study included the following.

- Study characteristics: Author(s), publication year, study design, and country of origin.
- UGV and radar technology details: Type of UGV, specific radar technology used, and the purpose of the radar integration.
- Outcomes: Key findings related to radar advancements, challenges in integration, and potential innovations.
- Risk of bias: Information on study limitations, including sample size, study design, and potential conflicts of interest.

### 2.6. Risk of Bias Assessment

The risk of bias in individual studies was assessed by the primary researcher through a qualitative evaluation of study methodologies. This assessment considered factors such as study design, data collection methods, and potential conflicts of interest, which are particularly relevant to the diverse types of studies included in this review. Any uncertainties encountered were discussed to ensure a reliable evaluation of potential biases in the included studies.

### 2.7. Summary Measures and Synthesis of Results

Quantitative data were synthesized where applicable; however, the focus of this review was primarily on qualitative synthesis, structured around key themes identified during data extraction. Thematic analysis was employed to interpret and summarize the findings, providing a comprehensive overview of the state of radar technologies in UGVs. This approach allowed for a detailed examination of advancements and challenges without relying on statistical measures like effect sizes or confidence intervals (CIs).

## 3. Results

### 3.1. Trends and Trajectories in Radar Technology Research: A Decade of Innovations and Applications (2014–2023)

**Figure 1:**
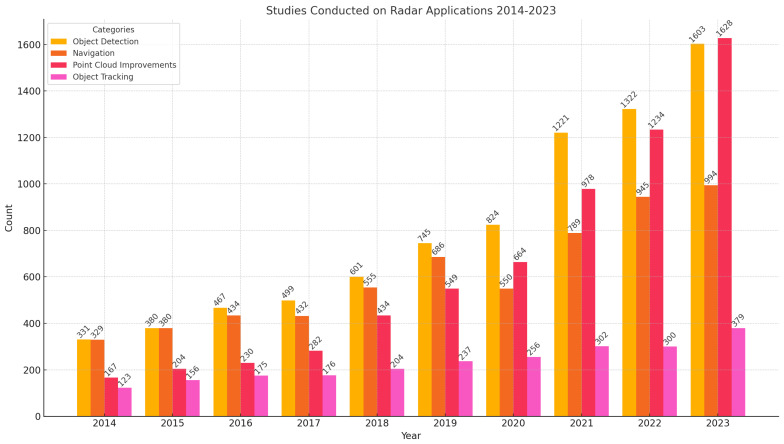
Annual research publications in radar applications from 2014 to 2023, categorized by application areas—object detection, point-cloud improvements, navigation, and object tracking.

In recent years, radar technology has experienced significant advancements, propelled by intensified research efforts across multiple domains. As illustrated in the accompanying chart, Figure 1, which categorizes the annual publication volume according to IEEE Xplore from 2014 to 2023 into four primary radar application areas (object detection, point-cloud improvements, navigation, and object tracking), there is evident, robust growth in scholarly output. This growth not only underscores the expanding capabilities of radar systems but also highlights the evolving intricacies and applications of radar technologies in modern contexts. This analysis investigates radar application trends, offering insights into the trajectories of radar technology development and its increasingly pivotal role across diverse sectors such as automotive, defense, and public safety. These patterns were examined with the aim of elucidating the technological evolution and industry shifts in radar-related research.

### 3.2. Annual Growth Analysis

This section offers an annual growth analysis of radar technology research, as shown in Figure 1. The analysis details year-over-year changes, identifying periods of significant growth and stability across each category. While this approach highlights overall trends and pivotal shifts in radar technology research, it is limited in scope and does not delve into the causative factors behind these trends or their broader industry implications.

- Object detection: Steady growth was observed from 2014 to 2023, with a total of 8993 studies in this period and particularly notable jumps in recent years (2021 to 2023). The largest increase occurred between 2022 and 2023, from 1322 to 1603 publications, which indicates increased interest or breakthroughs in technology relevant to object detection using radar.
- Point-cloud improvements: This category shows consistent growth, with a total of 6370 studies in this period and the most significant yearly increases around the later years, suggesting maturing technologies or enhanced applications of radar in generating and refining point clouds. The increase from 664 in 2020 to 1221 in 2021 stands out, possibly linked to advancements in 3D radar imaging technologies.
- Navigation: Navigation also shows strong growth, especially from 2020 onwards, underscoring the increasing reliance on radar technology for navigation systems, possibly driven by its applications in autonomous vehicles and mobile robotics. The total number of studies in this category is 6778.
- Object tracking: Object tracking underwent significant growth from 2020 onwards, peaking in 2023. This could be due to the expanding applications of radar in tracking systems for security, surveillance, and autonomous guidance systems. The total number of studies in this category is 2440.

### 3.3. Comparative Analysis

This section presents a comparative analysis of trends in radar technology research, as shown in Figure 1. The analysis highlights the fastest-growing category, emerging areas of interest, and consistent developments. While it offers an overview of dominant trends and emergent themes within the field, it does not capture every technological advancement or application across various industries.

- Fastest-growing category: Object detection has not only maintained the highest volume of publications but also shown the fastest growth, making it a key focus area within radar technology research. This growth is supported by significant advancements in radar-based object detection methods. For instance, consider the following.
  – Kim et al. (2020) demonstrated target classification using radar integrated with YOLO and SVM, achieving high accuracy in automotive applications [18].
  – Wang et al. (2021) introduced RODNet, a radar object detection network that fuses radar and camera data for improved 3D localization [19]. These examples illustrate the increasing attention toward radar-based object detection in autonomous navigation and collision-avoidance systems.
- Emerging interest: Point-cloud improvements show the largest percentage increase in publications year-over-year, particularly in 2021. This trend suggests that new methods or significant advancements in this area are gaining traction. Examples include the following.
  – Chen et al. (2023) developed methods for fusing LiDAR and radar point clouds to enhance density and accuracy, crucial for navigation in unstructured environments [20].
  – Khushaba et al. (2022) explored improvements in radar material classification, highlighting the role of detailed point clouds in detecting and identifying diverse environmental objects [21]. These innovations emphasize the growing importance of enriched point-cloud data for precision navigation and mapping applications.
- Consistent development: Navigation exhibits steady, linear growth in publications, reflecting ongoing the integration of radar technologies in transportation and logistics. Key contributions include the following.
  – Fritsche et al. (2016) detailed the fusion of LiDAR and radar data for SLAM, enhancing autonomous navigation in harsh environments [22].
  – Liu et al. (2022) investigated radar–camera fusion for robust target tracking in challenging weather, showcasing radar’s role in reliable navigation systems [23]. These examples highlight how radar technology continues to evolve, supporting autonomous navigation across diverse industries.

### 3.4. Overview of Included Studies

A comprehensive search across academic databases identified numerous studies. After inclusion and exclusion criteria were applied, 54 studies met the criteria and were included in the final review.

In the systematic examination of radar technology applications in UGVs, the diverse range of research areas is apparent. Object/obstacle detection leads with 19 studies, highlighting the emphasis on enhancing UGVs’ ability to identify objects via radar. Close behind, point-cloud improvement and navigation, with 14 and 12 studies, respectively, showcase interests in advancing environmental sensing and navigation. Meanwhile, object tracking, covered in nine studies, underscores the focus on safe navigation considerations in autonomous system deployment.

### 3.5. Advancements in Radar Technologies

Thematic analysis revealed key advancements in radar technologies applied to UGVs, categorized into four main themes:

- Theme 1: sensor resolution and accuracy—Studies emphasized advancements in radar sensor resolution, enhancing accuracy in detecting and tracking objects in various environmental conditions. For instance, Yamada et al. (2017) showcased a novel radar design that achieved unprecedented resolution in challenging weather conditions [24].
- Theme 2: integration with other sensor modalities—Integration with other sensor modalities, such as LiDAR and cameras, was a recurrent theme, contributing to comprehensive perception capabilities. Yang and Lho (2009) demonstrated successful integration strategies for enhancing UGV perception in diverse environments [1].
- Theme 3: real-time processing and decision-making—Advancements in real-time signal processing and decision-making algorithms were highlighted. Hongnan et al. (2022) proposed an innovative approach to on-board processing, significantly reducing latency in decision-making for embedded graphical processing unit GPU [25].
- Theme 4: adaptive and learning capabilities—Some studies explored radar systems with adaptive and learning capabilities, improving machine learning performance based on experience. Zhuangzhuang et al. (2016) implemented machine learning algorithms to enable radar point clouds to adapt to learning environmental conditions [26].

### 3.6. Challenges Addressed in the Literature

The literature identified several challenges associated with the integration of cutting-edge radar technologies with UGVs:

- Challenge 1: limited range in adverse weather conditions—Radar technologies are renowned for their reliability under challenging environmental conditions such as fog, rain, and snow. However, heavy precipitation and dense atmospheric disturbances can attenuate radar signals, particularly at higher frequencies like mmWave (77 GHz). This limitation reduces the effective detection range of radar in extreme weather. For instance, Xiong et al. (2022) investigated robust depth estimation methods by combining radar with RGB images to enhance performance in foggy environments [27].
  Advancements in radar signal processing, such as adaptive beamforming and wavelength-specific optimizations, have shown promise in mitigating these limitations. For example, consider the following.
  – Shorter wavelengths: mmWave radar excels in urban environments and moderate weather conditions but suffers from attenuation in heavy precipitation.
  – Longer wavelengths: Ku-band radar (12–18 GHz) provides better penetration through rain and fog, ensuring reliable long-range detection in adverse weather.
  These innovations highlight the importance of selecting the appropriate radar frequency and employing multi-sensor fusion techniques to address range limitations under extreme conditions.
- Challenge 2: complexities in data fusion—Studies highlighted challenges in effectively fusing radar data with data from other sensors, requiring sophisticated data fusion techniques. Chen et al. (2023) proposed a novel data fusion model to address challenges in integrating radar and LiDAR data [20].
- Challenge 3: implementing SLAM—SLAM is the computational process enabling a vehicle to construct or update a map of an unknown environment while simultaneously determining its location within this map. As Hong et al. mentioned in their research [28], while radar technologies offer robust sensing capabilities, especially in adverse weather conditions or where optical systems may falter, their application in SLAM for UGVs in rural environments introduces specific challenges such as dealing with sparse data, overcoming the effects of free space generation and natural clutter, extracting and associating features effectively, integrating diverse sensor fusion techniques, and ensuring system robustness against environmental variability.
- Challenge 4: obstacle detection and classification—While radar technologies excel in long-range detection and resilience to adverse weather, accurately identifying and classifying obstacles in diverse natural terrains remains a significant challenge. Natural environments introduce a wide variety of obstacles, ranging from small rocks and bushes to large boulders and fallen trees. Radar signals often scatter unpredictably off these surfaces, complicating object classification.
  To address this, advanced algorithms and signal processing techniques have been developed.
  – Deep learning models: Neural networks, such as those employed by Xiong et al. (2022), leverage radar data to improve obstacle classification accuracy in complex terrains [27].
  – Sensor fusion: Combining radar with LiDAR and cameras enhances the ability to distinguish objects of different sizes and textures, ensuring safer navigation through unstructured environments.
  Additionally, innovations in radar hardware, such as high-resolution imaging radar and polarization-sensitive radars, allow for better discrimination of objects based on their material properties and shapes. These advancements are critical for ensuring safe navigation in unpredictable terrains and overcoming the limitations of radar systems in obstacle detection.

**Figure 2:**
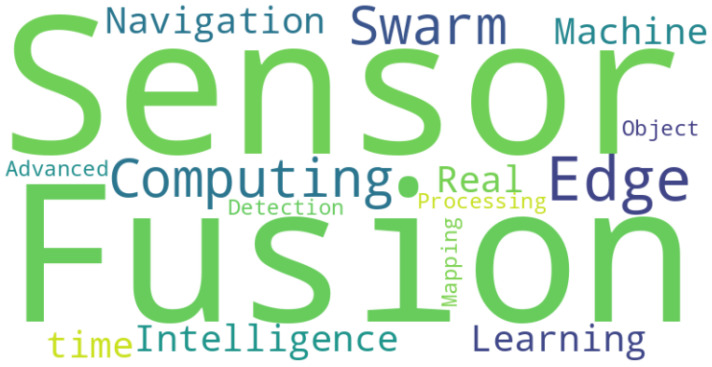
Key terms associated with sensor fusion technologies and related concepts discussed in the literature.

The word cloud in Figure 2 visually highlights the key terms frequently associated with sensor fusion technologies and related concepts discussed in the literature, such as computing, edge, swarm, machine learning, and real-time processing. These terms underscore the main themes and focus areas in current research and development.

### 3.7. State-of-the-Art Radar Applications to UGVs

Radar technologies for UGVs cater to a wide range of applications, including self-driving vehicles, environmental exploration, and disaster response. While the diverse use cases for UGVs (e.g., military surveillance, transportation, and indoor operations) entail different requirements, this manuscript primarily focuses on radar technologies for navigation, obstacle detection, and environmental mapping in non-military and unstructured environments. Specific military applications such as stealth countermeasures, DRFM, or quantum radar are beyond the scope of this work. To eliminate any confusion, the discussion is centered on radar’s role in enabling robust sensing and navigation for UGVs across general civilian and industrial use cases.

Radar systems used in UGVs employ a variety of advanced techniques to overcome challenges such as environmental clutter and limited range in adverse conditions. Key innovations include the following.

- FMCW radar: Widely adopted for its high-resolution (~2 cm) object detection capabilities, FMCW radar enables UGVs to measure velocity and range (up to 250 m) simultaneously, making it ideal for real-time collision avoidance in self-driving vehicles and off-road navigation [18,29,30].
- SAR: SAR generates high-resolution (below 10 cm) environmental maps and is particularly effective in agricultural and forestry applications where detailed terrain mapping is crucial. Its ability to operate reliably in low-visibility environments ensures effective performance in disaster response scenarios [24,25].
- Sensor fusion: Radar systems are often integrated with LiDAR and cameras to address the limitations of individual sensors. For instance, sensor fusion algorithms enable the detection of small objects at close range while maintaining long-range (100+ m) detection accuracy, making radar particularly valuable in autonomous navigation [20,22].
- Wavelength-specific applications: Different radar wavelengths (e.g., mmWave at 77 gigahertz (GHz) for short-range (up to 50 m), high-resolution detection and Ku-band (12–18 GHz) for long-range (up to 20 km) sensing) are employed, depending on the application requirements. For example, shorter wavelengths excel in urban obstacle detection, while longer wavelengths are effective in cluttered rural environments [10,21,24].

To demonstrate radar’s adaptability, the following real-world scenarios highlight its utility across various contexts:

- Self-driving vehicles: Radar systems are critical for detecting other vehicles, pedestrians, and obstacles in urban environments. With their capability to perform in adverse weather and low-light conditions, radars ensure collision avoidance and reliable autonomous operation [18,23].
- Disaster response: In search-and-rescue missions, radar systems help UGVs detect victims through smoke, debris, or snow. For instance, radar’s ability to penetrate environmental occlusions is unmatched in identifying objects obscured for optical and LiDAR sensors [30,31].
- Agricultural and forestry applications: UGVs equipped with radar systems navigate rough terrains, avoiding obstacles like fallen trees, rocks, and farming equipment. In forestry, radar assists in mapping dense vegetation, ensuring operational efficiency and safety [11,12].
- Indoor navigation: For warehouse or industrial UGVs, radar provides spatial awareness in GPS-denied environments, detecting shelving, equipment, and dynamic obstacles like personnel. Radar’s short-range, high-resolution capabilities are particularly effective in such settings [2,9,14].

Radar offers several distinct advantages over other sensing technologies, which make it indispensable for UGV operations:

- Resilience to adverse conditions: Unlike optical and LiDAR sensors, radar performs reliably in fog, rain, and snow, ensuring uninterrupted operations in adverse environments [21,23,32].
- Long-range detection: Radar excels in detecting objects at a distance, an essential feature for high-speed autonomous vehicles and long-range surveillance applications [22,30,33].
- Clutter management: Advanced algorithms mitigate clutter caused by environmental reflections, allowing radar to function effectively in dense, dynamic terrains [18,34,35].

The literature suggested several potential innovations in the application of radar technologies to UGVs: point-cloud improvements, object detection, navigation, mapping, slam, material classification, and obstacle detection.

Point-cloud data derived from radar systems play a critical role in enhancing spatial accuracy for UGVs. Accuracy requirements vary, depending on the application.

- Object detection: Precision levels of 1–2 cm are typically required for detecting small objects, such as debris or tools, ensuring reliable obstacle identification and safe navigation in cluttered environments [18,19].
- Mapping and SLAM: Autonomous navigation requires accurate spatial data to maintain localization and path planning, particularly in GPS-denied environments. For instance, centimeter-level accuracy is essential for high-resolution mapping and SLAM performance [22].

The accuracy of radar-generated point clouds is influenced by several factors.

- Wavelength: Shorter wavelengths, such as mmWave radar at 77 GHz, provide higher resolution but are more susceptible to attenuation and noise in adverse conditions. Longer wavelengths, such as Ku-band, offer improved penetration through clutter but at the cost of reduced spatial resolution [21,36].
- Environmental clutter: Dense vegetation, rain, or snow can scatter radar signals, reducing the fidelity of point cloud data. Advanced signal processing techniques, including polarization and beamforming, are employed to mitigate these effects [10].
- Hardware limitations: Sensor design, such as antenna array size and signal bandwidth, directly impacts resolution and accuracy [24].

Recent innovations have significantly improved radar point-cloud quality.

- High-bandwidth radar: Systems with larger signal bandwidths achieve finer resolutions, enabling precise material classification and obstacle detection [21].
- Sensor fusion: Integrating radar with LiDAR and camera data enriches point cloud detail, compensating for radar’s limitations in texture detection [20].

These advancements ensure that UGVs can detect obstacles, navigate unstructured environments, and interact safely with their surroundings, particularly in scenarios demanding high spatial accuracy.

#### 3.7.1. Object Detection

The field of radar-based object detection, coupled with material and obstacle recognition using UGV equipped with radar and machine learning, has seen remarkable growth and innovation. Recent advancements demonstrate radar’s robustness in detecting obstacles in unstructured environments, even under challenging conditions such as poor visibility and adverse weather [6,17]. Radar technology, especially UGV radar, excels in such scenarios, providing reliable obstacle detection and navigation capabilities [3].

Moreover, the integration of radar with LiDAR and other sensors further enhances object detection accuracy. Studies have shown that radar–LiDAR fusion significantly improves environmental perception, particularly in complex terrains [37]. Additionally, techniques like synthetic data generation and cross-modal supervision using auxiliary sensor data have enhanced radar-based machine learning models, enabling better training and reliability for object detection [16]. These advancements underscore radar’s pivotal role in autonomous UGV applications.

Advancements in neural network architectures tailored to radar data have opened new possibilities in enhancing both point cloud accuracy and object detection capabilities. For instance, the work by Shi et al. [38] introduced sophisticated models that are specifically optimized to process radar-generated data, leading to significant improvements in the detection and classification of objects, even in complex environments. These neural networks are designed to effectively handle the unique characteristics of radar signals, such as noise and variability, providing more robust performance in real-world scenarios.

In parallel, Khushaba et al. [21] employed deep wavelet-scattering transforms to push the boundaries of material classification using radar reflections. This method captures detailed features from radar signals, transforming raw data into structured representations that emphasize semantic information. By leveraging this approach, their model achieves high precision in classifying different materials based on subtle variations in radar reflections, which is particularly valuable for tasks requiring fine-grained object recognition.

Both techniques exemplify the shift towards more sophisticated signal processing methods in radar technology. By structuring raw radar data into formats that incorporate global semantic features, these models not only refine object detection but also enhance the reliability of confidence predictions. This combination of precision and reliability marks a substantial step forward in radar-based perception systems, enabling more accurate and dependable performance in autonomous navigation and other critical applications.

However, most of these research experiments have been tested under controlled laboratory conditions, focusing on non-mixed materials. While the results are promising, the real-world applicability, especially in mixed material environments for navigation and obstacle detection, remains to be fully proven. The classification of mixed materials is still in its early stages, suggesting a cautious approach towards integrating these technologies into existing projects. Continuous monitoring of developments in this field is recommended before making any integration decisions [21,36].

Advancements also include methodologies to overcome the shortage of accurately labeled data, with Wang et al. introducing synthetic data generation and cross-modal supervision that leverages auxiliary data from cameras and lidar to enrich radar data annotations [19]. This significantly improves the training of machine learning models for more reliable object detection.

Moreover, ongoing research continues to refine radar signal processing and target recognition, addressing specific challenges in millimeter-wave (mmWave) radar such as interference mitigation, enhanced height estimation, and robust algorithms for complex environments [30]. The exploration of new frequency bands, such as the W-band (75–110 GHz) and the Ka-band (26.5–40 GHz), is gaining attention due to their potential to improve spatial resolution and reduce interference in cluttered environments. Additionally, the Ku-band (12–18 GHz) is being utilized for long-range detection in adverse weather conditions. These advancements, combined with innovative antenna designs, promise to further enhance mmWave radar capabilities, ensuring precise and dependable object detection and material classification.

The intersection of radar technology and machine learning not only supports enhanced performance in adverse conditions but also drives advancements in various sectors. Frequency-modulated continuous-wave (FMCW) radar and mmWave radar, known for their high precision and ability to operate in challenging environments, have become crucial in enhancing object detection and navigation in UGVs. Synthetic-aperture radar (SAR) plays a significant role in applications ranging from military intelligence to urban planning and crop evaluation, providing high-resolution imaging even through obstructions. Additionally, ultra-wideband (UWB) radar is emerging as a key technology for low-power, cluttered environment sensing. These developments underscore the broad potential and growing relevance of radar technology in modern applications, continually pushing the boundaries of what is possible in remote sensing, security, and environmental monitoring [39].

The key advancements in object and obstacle detection applications for UGVs are summarized in Table 1, illustrating the range of innovative approaches in this rapidly evolving field.

#### 3.7.2. Navigation

Radar-based localization benefits from multiple sensors in an array, improving coverage and redundancy for robust performance. Integration with high-definition maps and sensor fusion refines accuracy, enabling precise positioning in complex urban scenarios.

Recent research [29] focuses on mitigating multi-path effects, enhancing accuracy in urban environments, and developing algorithms for dynamic scenarios. Advancements like using convolutional neural network (CNN) advantages with fused camera data and advanced signal processing contribute to improved localization, applicable to offroad fields.

An innovative approach involves angular measurements and interferometric measurements to estimate obstacle height in the track [30]. By utilizing delayed phase information, accurate height estimation is achieved, providing crucial details for navigating dynamic terrains beyond traditional radar-range information.

Another study demonstrates operational applicability of radar-based SLAM [42] by leveraging high-resolution radar imaging, the system can produce detailed maps and accurately localize UGVs in environments where the traditional global positioning system (GPS) and vision-based systems fail. Advanced algorithms process the radar data to account for dynamic objects, ensuring robust performance even in challenging indoor settings. The results indicate a significant improvement in navigation accuracy and reliability, showcasing the potential of radar-based SLAM for complex indoor applications.

Hong et. al highlights the use of FMCW radar sensors for robust SLAM in various weather conditions. It employs graph-based matching algorithms and integrates radar data with IMU to enhance the system’s robustness and accuracy [43]. This approach allows for effective odometry and SLAM even in dynamic city environments. The integration of graph-based algorithms ensures that the SLAM system can handle the non-linearities and inconsistencies typically found in urban landscapes. Moreover, the use of IMUs provides additional stability and precision, which is crucial for maintaining accurate positioning in scenarios where radar data might be temporarily unreliable.

The key advancements in radar-based navigation applications for UGVs are summarized in Table 2, demonstrating the diverse approaches to improving localization, SLAM, and obstacle detection in both urban and off-road scenarios.

#### 3.7.3. Object Tracking

The latest research on autonomous driving leveraging radar technology to track objects marks a significant advancement in enhancing the situational awareness and safety of UGVs and other self-driving architectures. Radar is known for its reliability in various environmental conditions, and it is increasingly becoming a crucial sensor for tracking objects and ensuring precise navigation. One noteworthy avenue of exploration involves the integration of deep neural networks into radar-based perception systems. Deep neural networks (DNNs) excel at learning complex patterns and features from large datasets, making them well-suited for radar data interpretation. Neural network architectures are specifically developed to process radar imagery, enabling more advanced and accurate object detection and classification. The operation between radar and DNNs contributes to creating a robust perception framework crucial for the safe navigation of autonomous vehicles.

Additionally, particle filters, a probabilistic method for state estimation, are being employed in conjunction with radar imagery to enhance object tracking capabilities [44]. The integration of particle filters with radar data allows for dynamic tracking of objects, considering uncertainties and variations in real-world scenarios. This approach proves valuable in maintaining accurate object trajectories, especially in situations with complex interactions and changing environmental conditions.

Research over the past few years has highlighted several UGV radar applications for autonomous driving, including the development of postprocessing architectures for clustering and tracking multiple radar detections from one object, enhancing reliability in various driving maneuvers [45]. Kalman filter-based approaches are also prevalent, particularly for border security and surveillance systems, emphasizing radar’s critical role in accurate object tracking under diverse conditions [46]. Furthermore, real-time road-object detection and tracking methods that fuse LiDAR and radar data using unscented Kalman filters have been shown to improve precision in tracking objects such as bicycles, cars, and pedestrians [47].

The integration of LiDAR and mmWave radar has been explored to ensure safer UGV autonomous navigation and effective collision avoidance. Specifically, close proximity refers to detection ranges within 0.8–120 m, for which the accurate identification of small objects is critical for real-time navigation. Advanced sensor-fusion algorithms enable UGVs to detect, classify, and track objects [48]. This capability ensures that UGVs can safely navigate dynamic environments by promptly taking action based on fused sensor data.

Moreover, comparative studies of different backbones and object detector heads on radar-based object detection algorithms have demonstrated significant advancements. For example, integrating a YOLOv4 head with a modified residual network ResNet backbone has shown improved detection accuracy and robustness in diverse environmental conditions. These advancements achieve precision levels exceeding 66.3%, enabling reliable collision avoidance and enhanced operational safety across a wide range of scenarios [49].

The transformative impact of these radar advancements is summarized in Table 3, illustrating the range of innovative applications in object tracking for autonomous driving systems. These developments highlight radar technology’s capacity to adapt and excel in complex and dynamic environments, ensuring greater safety and efficiency for UGVs.

#### 3.7.4. Point-Cloud Improvements

Further enhancing the utility of point clouds, Chen et al. (2023) [20] have developed methods for the fusion of LiDAR and mmWave radar point clouds for environment sensing. This fusion not only improves the density and accuracy of the environmental data collected but also enhances the reliability of the vehicle’s perception systems in diverse atmospheric conditions, such as fog, heavy rain, and low-light environments. Additionally, the work by Xiong et al. (2022) on robust depth estimation in foggy environments by combining RGB images with mmWave radar demonstrates a sophisticated approach to maintaining accuracy in depth measurements when optical sensors are compromised. These conditions, referred to as “visually impaired”, highlight scenarios where visibility for traditional vision-based systems, such as cameras and LiDAR, is significantly reduced due to environmental factors. In such situations, mmWave radar’s ability to penetrate atmospheric occlusions ensures safer navigation and obstacle detection. Together, these developments represent a significant stride toward more reliable and versatile radar systems in automotive and other navigation-related applications.

The contributions of various studies to point-cloud improvements in radar technologies applied to UGVs are summarized in Table 4. This table outlines the key advancements in sensor fusion, depth estimation, and environmental perception, all contributing to the enhancement of UGV capabilities in complex terrains.

### 3.8. Enhancing Autonomy Through Radar Integration

Radar technology has emerged as a cornerstone for advancing autonomy across various domains, including defense, transportation, and exploration. By providing high-resolution, all-weather sensing capabilities, radar enables autonomous systems to operate reliably in complex and dynamic environments. By integrating radar with other sensor modalities, autonomous platforms achieve more advanced navigation, obstacle detection, and situational awareness. This section explores radar’s role across key applications, highlighting its contributions to the development of autonomous systems in defense, transportation, and exploration.

#### 3.8.1. Transportation Applications

Radar technology transforms the transportation sector during this period, enabling autonomous vehicle advancements. Ji et al. [55] combined radar with vision systems to improve object detection for autonomous driving. This radar-vision fusion approach was foundational for developing advanced driver assistance systems (ADASs) and autonomous driving technologies, setting a standard for subsequent work in the field.

Gordon et al. [56] emphasized radar’s role in enabling automated driving functionalities, such as enhanced steering systems for safety-critical maneuvers. This work highlighted radar’s ability to integrate with other sensory modalities to support semi-autonomous and fully autonomous vehicles.

Bayless et al. [57] discussed radar’s integration in connected vehicle ecosystems. Active radar systems were shown to significantly enhance road safety by enabling crash mitigation technologies. These contributions demonstrated radar’s capacity to not only enable autonomy but also improve overall vehicular safety and reliability.

#### 3.8.2. Defense Applications

Radar technology plays a critical role in bolstering defense-related autonomous systems. Djapic et al. [58] demonstrated how sonar and radar integration could enhance the situational awareness of autonomous surface vehicles. By fusing these technologies, the system achieved superior navigation and obstacle avoidance capabilities in mine countermeasure scenarios. This work paved the way for the development of robust autonomous systems for maritime defense.

Banks et al. [59] explored swarm intelligence in radar-equipped UAVs, focusing on air defense applications. By leveraging particle swarm algorithms, the study introduced a novel radar-guided system capable of coordinating UAV movements in complex air defense scenarios. This innovation underscores radar’s role in augmenting UAV capabilities for military applications.

In the broader context, Griffiths et al. [60] delved into bistatic radar’s potential for military applications. Their work detailed enhancements in radar cross-section analysis, essential for improving threat detection and mitigation in autonomous military systems. These advancements demonstrated radar’s pivotal contribution to real-time, reliable decision-making in defense systems.

#### 3.8.3. Exploration Applicaitons

Exploration missions benefit significantly from radar-based advancements, particularly in mapping and navigation. Farr et al. [61] detailed the Shuttle Radar Topography Mission (SRTM), a pioneering effort in global topographic mapping. This work laid the groundwork for autonomous planetary exploration systems by demonstrating radar’s ability to generate precise digital elevation models.

Underwater environments also witnessed innovations, with Villar et al. [62] showcasing radar-enhanced mapping systems for autonomous underwater vehicles. This approach allowed for detailed perception in challenging underwater terrains, facilitating exploration and monitoring tasks.

In urban contexts, Leonard et al. [63] advanced autonomous vehicle perception systems by integrating radar to navigate complex urban environments. This work underscored radar’s importance in addressing challenges such as dynamic obstacles and limited visibility.

Lastly, Le et al. [64] presented ultrawideband radar imaging for interior navigation, expanding radar’s applications to indoor environments. This study demonstrated radar’s utility in enabling autonomous systems to operate in GPS-denied areas, a critical capability for exploration missions.

## 4. Research Gaps in the State of the Art

Despite the richness of the literature, certain gaps were identified, suggesting areas for future research.

- Gap 1: adapting radar systems to diverse weather conditions—Existing research lacks a comprehensive understanding of how adverse weather conditions, including rain, snow, and fog, impact the performance of radar-based obstacle detection systems in rural/off-road environments.

A research gap is evident in the absence of comprehensive insights into techniques and algorithms for enhancing obstacle detection robustness under varying meteorological conditions. Future studies should focus on mitigating adverse weather effects on raw radar data and ensuring UGV navigation reliability.

- Gap 2: radar performance in vegetated environments—There is a limited understanding of how radar systems perform in environments with varying levels of vegetation density, and the influence of dense foliage on radar signals remains insufficiently explored.

A notable research gap exists in addressing challenges related to vegetation interference and proposing effective solutions to enhance obstacle detection reliability in vegetated terrains. Future research should conduct in-depth investigations into radar performance in such scenarios.

- Gap 3: obstacle classification in off-road conditions—Ongoing challenges persist in accurately classifying obstacles detected by radar in off-road conditions, including distinguishing between rocks, trees, holes, and other static objects.

Future research should delve into enhancing the accuracy of classifying detected obstacles, providing more detailed information for UGV decision-making during off-road navigation.

- Gap 4: radar performance in off-road environments—The existing literature indicates a gap in understanding how radar systems perform in off-road conditions, encompassing rugged terrains, gravel paths, and uneven surfaces.

Research should prioritize adapting radar-based obstacle detection algorithms to address the complexities of off-road environments, ensuring reliable performance in irregular and unpredictable terrains.

- Gap 5: radar–based terrain identification—The current literature lacks comprehensive explanations or methodologies on using existing radar technologies for specific terrain identification.

There is a significant need for studies that investigate the feasibility and effectiveness of using radar data to identify the nature of the terrain (solid ground, gravel, mud, etc.), which is crucial for safe UGV navigation, especially in scenarios where terrain type may impact vehicle stability.

- Gap 6: optimization of sensor fusion strategies—While sensor fusion is acknowledged for enhancing object detection, classification, and navigation accuracy, there is a research gap in optimizing fusion strategies, especially in complex scenarios.

Advanced research should concentrate on developing robust sensor fusion frameworks tailored to the unique challenges of UGV deployments, encompassing varying vegetation density, off-road conditions, and diverse weather scenarios.

## 5. Discussion and Conclusions

This systematic literature review has broadly examined the integration of advanced radar technologies in UGVs, highlighting their crucial role in advancing autonomy in various fields like defense, transportation, and exploration. Despite the significant progress in radar technology, integrating these innovations with UGVs has presented notable challenges. This exploration has shed light on state-of-the-art research, pinpointing advancements, addressing existing challenges, and suggesting directions for future studies. Through a rigorous and systematic approach, this review has synthesized existing knowledge, underscoring the advantages of radar technologies in enhancing UGV capabilities.

The findings reveal both the potential and the hurdles in fully realizing radar-enhanced UGV autonomy. Key advancements have improved sensor resolution, accuracy, and the integration with other sensors, fostering better navigation and perception. However, challenges such as limited radar range in adverse conditions, complexities in data fusion, and the intricacies of implementing effective SLAM strategies in non-urban environments have been identified as areas needing further exploration. Additionally, the review has pinpointed several gaps in the literature, especially the need for research focused on radar performance in vegetated environments, obstacle classification in off-road conditions, and the optimization of sensor fusion strategies.

Addressing these gaps is not just crucial for advancing radar technology in UGVs but also essential for the broader adoption of autonomous systems in real-world applications. Future research should aim at enhancing radar systems’ adaptability to diverse environmental conditions, improving obstacle detection and classification accuracy, and developing more sophisticated sensor fusion models. By overcoming these challenges, the next generation of UGVs equipped with cutting-edge radar technologies can achieve higher levels of autonomy, reliability, and operational efficiency, paving the way for their expanded use across a wider range of applications.

While this review highlights significant advancements in radar technology for UGVs, it also underscores the critical challenges that remain. By addressing the limitations in radar performance under adverse conditions, refining sensor fusion techniques, and improving obstacle detection capabilities, the potential for fully autonomous UGV systems can be greatly expanded. The following research questions were formulated to guide the future direction of research, aiming to enhance the integration of radar technologies with UGVs and overcome the current obstacles identified in this review.

**RQ1:** Recent advancements in radar technologies, such as high-resolution radar imaging, mmWave radar, and FMCW radar, have significantly enhanced UGV capabilities. These innovations improve sensor resolution and accuracy, enabling UGVs to detect and classify objects with higher precision. Enhanced radar signal processing techniques have also improved navigation and obstacle avoidance, allowing UGVs to map their surroundings more accurately and navigate through complex terrains with greater operational efficiency, even in adverse weather conditions.

**RQ2:** The integration of current radar technologies with machine learning and sensor fusion techniques has significantly improved the accuracy and reliability of object detection, navigation, and object tracking in UGVs. Machine learning algorithms, particularly deep learning models, are effectively trained to interpret radar data, leading to more precise object recognition and classification. Sensor fusion combines data from radar, LiDAR, cameras, and other sensors, enhancing the robustness of UGV perception systems. This multi-sensor approach mitigates the limitations of individual sensors, resulting in more accurate and reliable detection and tracking of objects, as well as improved navigation capabilities.

**RQ3:** Emerging trends and future directions in radar technology research for UGVs focus on improving performance, efficiency, and applicability. Notable trends include the development of advanced machine learning models specifically tailored to radar data, the exploration of novel radar frequency bands and antenna designs, and the miniaturization and cost reduction in radar components. Research is also directed towards radar-based SLAM and the integration of radar with emerging technologies such as edge computing and 5G connectivity. These advancements are expected to play a crucial role in enabling more autonomous and intelligent navigation systems.

The main limitation of the included studies is the lack of real-world testing. Most of the advancements were tested under controlled environments, limiting their applicability in complex terrains or adverse weather conditions. Additionally, many studies did not provide long-term testing data to assess the durability and reliability of the radar systems.

The review process was limited due to the exclusion of non-English studies, which may have omitted relevant research. Additionally, unpublished or industry-specific studies were not included, though they could provide different perspectives on radar technologies. The review also relied primarily on peer-reviewed publications, potentially overlooking cutting-edge but non-peer-reviewed work.

The integration of radar technologies into UGVs offers significant potential to enhance perception and navigation, improving the overall safety and operational efficiency of autonomous vehicles. However, limitations in current obstacle detection and data fusion systems suggest that practitioners should approach deployment with caution, particularly in complex or unstructured environments where performance may be compromised. From a policy perspective, it is essential to establish clear standards and guidelines for the use of radar technologies in autonomous systems, ensuring reliability across various environmental conditions. For future research, attention should be directed toward addressing gaps in radar performance under adverse weather conditions, optimizing sensor fusion strategies, and improving obstacle detection, especially in off-road and mixed-terrain environments. Additionally, exploring more advanced multi-sensor fusion techniques will be critical to enhancing the robustness and adaptability of UGV systems in challenging scenarios.

In conclusion, while radar technologies have made considerable strides in enhancing UGV autonomy, significant challenges remain that must be addressed to fully realize their potential. The next generation of UGVs will require continued advancements in radar resolution, data-fusion algorithms, and real-time processing capabilities to ensure robust performance across a range of environments. Future research should also focus on expanding the applicability of radar-based SLAM and improving obstacle classification accuracy in off-road conditions. By overcoming these barriers, radar technologies can play a transformative role in enabling UGVs to navigate complex, real-world environments with greater autonomy and reliability.

## Figures and Tables

**Table 1:** Summary of object/obstacle detection applications in radar technologies applied to UGVs.

Ref	Title	Author	Year	Short Analysis of the Main Outcomes (with Advantages and Disadvantages)
[17]	Autonomous Driving in Unstructured Environments: How Far Have We Come?	Min et al.	2024	A detailed exploration of the challenges and advancements in radar-based object detection for dynamic obstacles in unstructured environments. **Advantages:** Focuses on unstructured environments. **Disadvantages:** Limited experimental validations.
[7]	A Low-Cost Relative Positioning Method for UAV/UGV Coordinated Heterogeneous System Based on Visual-Lidar Fusion	Luo et al.	2023	Investigates radar and LiDAR integration for precise object detection and navigation in industrial and warehouse environments, improving UGV collision avoidance. **Advantages:** Low-cost and precise. **Disadvantages:** Requires controlled environments for optimal performance.
[23]	Robust Target Recognition and Tracking of Self-Driving Cars With Radar and Camera Information Fusion Under Severe Weather Conditions	Liu et al.	2022	Fusion techniques are utilized to enhance vehicle autonomy by combining radar and camera data, improving perception capabilities in challenging weather. **Advantages:** Works well under severe weather conditions. **Disadvantages:** Computationally intensive.
[21]	Radar-Based Materials Classification Using Deep Wavelet Scattering Transform: A Comparison of Centimeter vs. Millimeter Wave Units	Khushaba et al.	2022	A detailed comparison of radar technologies for material classification, employing the deep wavelet scattering transform to distinguish between materials. **Advantages:** High classification accuracy. **Disadvantages:** Limited to material-specific environments.
[30]	Obstacle Height Estimation through Vehicular Radar Interferometry	He et al.	2021	A method for estimating the height of obstacles using radar interferometry is presented, enhancing vehicular safety and navigation. **Advantages:** Accurate height estimation. **Disadvantages:** Limited applicability in real-time scenarios.
[39]	Radar based obstacle detection in unstructured scene	Li et al.	2021	A method using radar to identify and navigate around obstacles in unstructured environments. **Advantages:** Effective for unstructured terrains. **Disadvantages:** Limited in cluttered or vegetated areas.
[19]	RODNet: A Real-Time Radar Object Detection Network Cross-Supervised by Camera-Radar Fused Object 3D Localization	Wang et al.	2021	The synergistic use of radar and camera data for enhancing real-time object detection capabilities is explored. **Advantages:** High precision in diverse environments. **Disadvantages:** Requires advanced fusion algorithms.
[32]	Perception and sensing for autonomous vehicles under adverse weather conditions: A survey	Zhang et al.	2021	A comprehensive overview of challenges and technologies for vehicle perception in adverse weather is provided. **Advantages:** Focus on adverse conditions. **Disadvantages:** Limited real-world examples.
[40]	On the Use of Low-Cost Radars and Machine Learning for In-Vehicle Passenger Monitoring	Abedi et al.	2020	Highlights radar applications for in-vehicle passenger monitoring, improving safety through machine learning. **Advantages:** Low cost. **Disadvantages:** Limited to specific use cases.
[41]	RadarNet: Exploiting Radar for Robust Perception of Dynamic Objects	Yang et al.	2020	Integrates radar with computer vision for detecting dynamic objects in complex environments. **Advantages:** Effective in complex environments. **Disadvantages:** Computational demands.
[18]	Target Classification Using Combined YOLO-SVM in High-Resolution Automotive FMCW Radar	Kim et al.	2020	A novel YOLO-SVM approach for target classification is proposed, improving reliability in automotive applications. **Advantages:** High target classification accuracy. **Disadvantages:** Computational overhead.
[38]	PointRCNN: 3D Object Proposal Generation and Detection From Point Cloud	Xiaogang et al.	2019	Introduces a deep learning framework for 3D object proposals from point clouds. **Advantages:** Accurate 3D detection. **Disadvantages:** Requires high computational resources.
[36]	Material Classification using 60-GHz Radar and Deep Convolutional Neural Network	Weiß et al.	2019	Enhances material classification using high-frequency radar signals with deep learning. **Advantages:** High-frequency accuracy. **Disadvantages:** Limited scalability.
[5]	Imaging radar for navigation and surveillance on an autonomous unmanned ground vehicle capable of detecting obstacles obscured by vegetation	Gusland et al.	2019	A radar solution that detects obstacles hidden by natural barriers. **Advantages:** Penetrates vegetation. **Disadvantages:** Reduced resolution in dense areas.
[31]	Through-Wall Human Pose Estimation Using Radio Signals	Zhao et al.	2018	Radio signals are used for human pose estimation through obstacles. **Advantages:** Penetrates walls. **Disadvantages:** Limited to static scenarios.
[34]	VoxelNet: End-to-End Learning for Point Cloud Based 3D Object Detection	Zhou et al.	2018	A deep learning architecture for 3D object detection from point clouds is developed. **Advantages:** High accuracy. **Disadvantages:** High computational cost.
[10]	Learned Ultra-Wideband Radar Sensor Model for Augmented LiDAR-Based Traversability Mapping in Vegetated Environments	Ahtiainen et al.	2015	Explores radar-LiDAR fusion for enhanced obstacle detection in vegetated terrains. **Advantages:** Effective in vegetation. **Disadvantages:** Limited terrain diversity.
[11]	Towards LIDAR-RADAR Based Terrain Mapping	Guerrero et al.	2015	Explores terrain mapping using radar and LiDAR fusion, enhancing obstacle detection capabilities. **Advantages:** Improved mapping accuracy. **Disadvantages:** Limited scalability to urban environments.
[1]	Sensor fusion for obstacle detection and its application to an unmanned ground vehicle	Yang et al.	2009	Enhances obstacle detection by combining data from multiple sensors. **Advantages:** High reliability through fusion. **Disadvantages:** Requires multiple hardware components.

**Table 2:** Summary of navigation applications in radar technologies applied to UGVs.

Ref	Title	Author	Year	Short Analysis of the Main Outcomes (with Advantages and Disadvantages)
[7]	A Low-Cost Relative Positioning Method for UAV/UGV Coordinated Heterogeneous System Based on Visual-Lidar Fusion	Luo et al.	2023	Explores cost-effective sensor fusion techniques, combining visual and LiDAR data to improve navigation accuracy in coordinated UAV and UGV systems. **Advantages:** Low-cost approach with high accuracy. **Disadvantages:** Limited robustness in adverse environments.
[16]	Challenges and Solutions for Autonomous Ground Robot Scene Understanding and Navigation in Unstructured Outdoor Environments: A Review	Rassau et al.	2023	Discusses radar technologies for autonomous navigation in unstructured terrains, emphasizing obstacle detection and path planning. **Advantages:** Highlights critical challenges in unstructured environments. **Disadvantages:** Lacks experimental validation.
[42]	Indoor Radar SLAM for Vision and GPS Denied Environments	Li et al.	2022	The operational applicability of radar-based SLAM technology for indoor use is demonstrated, effectively handling dynamic objects and providing reliable localization in GPS-denied environments. Radar data is used in conjunction with SLAM algorithms to achieve accurate positioning. **Advantages:** Reliable in GPS-denied environments. **Disadvantages:** Less effective in larger-scale operations.
[28]	RadarSLAM: A robust simultaneous localization and mapping system for all weather conditions	Hong et al.	2022	A robust SLAM system that operates effectively across diverse weather conditions using radar technology is developed. The methodology involves integrating radar data with other sensor inputs to maintain localization accuracy. **Advantages:** Weather-agnostic localization performance. **Disadvantages:** Computationally demanding integration process.
[29]	Frequency Modulated Continuous Wave Radar-Based Navigation Algorithm using Artificial Neural Network for Autonomous Driving	Valtl et al.	2021	An algorithm that leverages neural networks to improve the accuracy of radar-based navigation systems is implemented. Frequency modulated continuous wave (FMCW) radar data is processed using artificial neural networks to enhance navigation precision. **Advantages:** High precision in navigation algorithms. **Disadvantages:** Dependency on extensive training data.
[35]	Radar Visual Inertial Odometry and Radar Thermal Inertial Odometry: Robust Navigation even in Challenging Visual Conditions	Doer et al.	2021	Robust navigation in challenging visual conditions is investigated. Novel odometry methods that maintain accurate navigation when visual signals are compromised are examined. The integration of radar with visual and thermal data ensures reliable odometry. **Advantages:** Reliable in low-visibility conditions. **Disadvantages:** Higher implementation complexity.
[43]	Radar SLAM: A Robust SLAM System for All Weather Conditions	Hong et al.	2021	Graph-based matching algorithms are implemented and radar data is integrated with IMUs to enhance the system’s robustness and accuracy. The SLAM system’s performance in various weather conditions is evaluated. **Advantages:** Strong performance under diverse weather conditions. **Disadvantages:** Limited scalability for large environments.
[22]	Fusing LiDAR and Radar Data to Perform SLAM in Harsh Environments	Fritsche et al.	2016	Combines LiDAR and radar sensor data to enhance SLAM performance, specifically in unstructured and visually impaired conditions. **Advantages:** Enhanced SLAM in harsh conditions. **Disadvantages:** Requires high computational resources.
[10]	Learned Ultra-Wideband Radar Sensor Model for Augmented LiDAR-Based Traversability Mapping in Vegetated Environments	Ahtiainen et al.	2015	Proposes radar sensor models for terrain navigation, particularly in vegetated or cluttered outdoor environments, enhancing traversability mapping and obstacle avoidance. **Advantages:** Effective in dense vegetation. **Disadvantages:** Limited generalization to non-vegetated terrains.
[14]	LiDAR-Aided Indoor Navigation System for UGVs	Atia et al.	2015	Introduces radar-LiDAR sensor fusion for improving indoor navigation systems in GPS-denied environments, ensuring precise localization and mapping. **Advantages:** Highly precise indoor navigation. **Disadvantages:** Inapplicable to outdoor settings.
[2]	Knowledge-based indoor positioning based on LiDAR aided multiple sensors system for UGVs	Chen et al.	2014	A system that enhances indoor positioning for UGVs using multiple sensor inputs is discussed. LiDAR data is combined with other sensors to improve positioning accuracy. **Advantages:** Enhanced accuracy through sensor fusion. **Disadvantages:** Reliance on high-cost hardware.
[15]	A fusion study of the FAVS sensors suite	Villers et al.	2007	The integration of various sensor technologies to enhance data accuracy and reliability in fusion systems is analyzed. Multiple sensor data are fused to improve overall system performance. **Advantages:** Improved system reliability through fusion. **Disadvantages:** Limited to small-scale environments.

**Table 3:** Summary of object tracking applications in radar technologies applied to UGVs.

Ref	Title	Author	Year	Short Analysis of the Main Outcomes (with Advantages and Disadvantages)
[49]	Comparative Analysis of mmWave Radar-based Object Detection in Autonomous Vehicles	Sharifisoraki et al.	2024	The effectiveness of different radar technologies and machine learning algorithms in detecting objects is evaluated, aiming to optimize sensors for better environmental perception in autonomous vehicles. **Advantages:** High detection accuracy with machine learning algorithms. **Disadvantages:** Limited evaluation under adverse weather conditions.
[48]	Lidar-mmWave Radar Fusion for Safer UGV Autonomous Navigation with Collision Avoidance	Robsrud et al.	2023	The integration of lidar and mmWave radar data is demonstrated to enhance obstacle detection and avoidance, ensuring safer navigation for UGVs. **Advantages:** Improved obstacle detection with sensor fusion. **Disadvantages:** Increased computational cost and complexity.
[46]	Kalman Filter-Based Suspicious Object Tracking for Border Security and Surveillance Systems using Fixed Automotive Radar	Park et al.	2023	The application of Kalman filtering techniques to improve the accuracy and reliability of object tracking systems in security-sensitive environments are detailed. **Advantages:** Reliable object tracking for surveillance applications. **Disadvantages:** Performance is limited by radar resolution.
[9]	Real-Time 3D-LiDAR, MMW Radar, and GPS/IMU Fusion for UGV Detection and Tracking in Unstructured Environments	Li et al.	2021	A real-time framework combining 3D-LiDAR, mmWave radar, and GPS/IMU for improved detection and tracking in unstructured environments is introduced, highlighting its utility for autonomous navigation. **Advantages:** High robustness in unstructured environments. **Disadvantages:** Dependency on precise sensor calibration.
[44]	Combined Object Detection and Tracking on High Resolution Radar Imagery for Autonomous Driving Using Deep Neural Networks and Particle Filters	Stroescu et al.	2020	Enhanced object detection and tracking capabilities using advanced radar imaging and computational techniques are demonstrated. **Advantages:** Improved detection accuracy with neural networks. **Disadvantages:** Computationally expensive for real-time use.
[47]	Road-objects tracking for autonomous driving using lidar and radar fusion	Farag et al.	2020	The fusion of lidar and radar data to improve the tracking of road objects is examined, contributing to safer and more reliable autonomous driving technologies. **Advantages:** Enhanced tracking performance with sensor fusion. **Disadvantages:** Fusion algorithms require extensive testing in real-world conditions.
[12]	A Survey on Sensor Technologies for Unmanned Ground Vehicles	Liu et al.	2020	Discusses the use of radar in tracking dynamic obstacles and improving UGV navigation. Insights on integrating radar with other sensors for enhanced obstacle tracking are provided. **Advantages:** Provides comprehensive insights into sensor technologies. **Disadvantages:** Limited focus on practical implementation details.
[45]	Radar Based Object Detection and Tracking for Autonomous Driving	Manjunath et al.	2018	The implementation of radar technology to enhance object detection and tracking capabilities is discussed, crucial for autonomous vehicle safety and efficiency. **Advantages:** Effective for tracking in real-time environments. **Disadvantages:** Limited validation under extreme environmental conditions.
[50]	A scanning FMCW-radar system for the detection of fast moving objects	Shoykhetbrod et al.	2014	A radar system optimized for high-speed object detection is described, enhancing response times and accuracy in dynamic environments. **Advantages:** High-speed object detection capability. **Disadvantages:** Limited application for slow-moving or stationary objects.

**Table 4:** Summary of point-cloud improvements in radar technologies applied to UGVs.

Ref	Title	Author	Year	Short Analysis of the Main Outcomes (with Advantages and Disadvantages)
[4]	A Comprehensive Survey of UGV Terrain Traversability for Unstructured Environments and Sensor Technology Insights	Beycimen et al.	2023	Surveys advancements in radar-based point-cloud improvements, particularly for terrain traversability and object detection in unstructured environments. **Advantages:** Comprehensive insights into UGV navigation challenges. **Disadvantages:** Limited practical applications presented.
[20]	Fusion of LiDAR and mmWave Radar Point Clouds for Environment Sensing	Chen et al.	2023	The combination of data from LiDAR and radar to enhance environmental perception and object detection is examined. Sensor fusion techniques are used to integrate point cloud data from both sources. **Advantages:** Improved accuracy and perception through fusion. **Disadvantages:** Higher computational cost due to fusion complexity.
[51]	Sparse-to-Dense Matching Network for Large-Scale LiDAR Point Cloud Registration	Lu et al.	2023	A network that improves the accuracy of point cloud registration is developed, essential for detailed environmental mapping. Sparse-to-dense matching techniques are employed to achieve better registration accuracy. **Advantages:** High registration accuracy for mapping. **Disadvantages:** Performance may degrade in highly cluttered environments.
[52]	Autonomous Propulsion for a GPR-UGV	Eskilsson	2022	Introduces ground-penetrating radar (GPR) integrated with UGV propulsion systems to enhance navigation in rugged and unstructured terrains. **Advantages:** Effective in detecting subsurface obstacles. **Disadvantages:** Limited performance in non-rugged terrains.
[27]	Robust Depth Estimation in Foggy Environments Combining RGB Images and mmWave Radar	Xiong et al.	2022	A method that integrates radar and optical images to improve depth estimation under impaired visibility conditions is described. RGB images and mmWave radar data are combined to enhance depth perception. **Advantages:** Reliable in adverse weather conditions. **Disadvantages:** Performance depends on accurate RGB image alignment.
[25]	Research on Real-time Imaging Method of Airborne SAR Based on Embedded GPU	Tian et al.	2022	A method for enhancing the real-time imaging capabilities of synthetic aperture radar using advanced processing technologies is explored. Embedded GPUs are utilized to process SAR data more efficiently. **Advantages:** High processing speed. **Disadvantages:** Hardware reliance increases system cost.
[9]	Real-Time 3D-LiDAR, MMW Radar, and GPS/IMU Fusion for UGV Detection and Tracking in Unstructured Environments	Li et al.	2021	A real-time sensor fusion framework combining 3D-LiDAR, radar, and GPS/IMU to improve UGV detection and tracking in unstructured environments. **Advantages:** Robust tracking in unstructured terrains. **Disadvantages:** Requires precise sensor calibration.
[53]	Inverse synthetic aperture radar imaging exploiting dictionary learning	Hu et al.	2018	Advanced processing techniques to refine radar imaging are introduced, significantly improving the resolution and clarity of radar-derived images. Dictionary learning is employed to enhance image quality. **Advantages:** Superior image clarity. **Disadvantages:** Processing-intensive for real-time applications.
[24]	High-resolution 2D SAR imaging by the millimeter-wave automobile radar	Yamada et al.	2017	A technique for producing high-resolution radar images is detailed, improving the detection and classification of objects in automotive applications. Millimeter wave radar is used to achieve high-resolution imaging. **Advantages:** High-resolution imaging for automotive applications. **Disadvantages:** Limited to short-range applications.
[26]	Adaptive learning rate CNN for SAR ATR	Zhuangzhuang et al.	2016	Adaptive learning techniques to improve the performance of convolutional neural networks in radar target recognition are investigated. CNNs with adaptive learning rates are used for automatic target recognition (ATR). **Advantages:** Improved target recognition accuracy. **Disadvantages:** Requires significant training data.
[22]	Fusing LiDAR and Radar Data to Perform SLAM in Harsh Environments	Fritsche et al.	2016	A technique that combines data from multiple sensors to enhance SLAM reliability in adverse conditions is described. LiDAR and radar data are fused to improve SLAM performance. **Advantages:** Enhanced SLAM reliability in harsh conditions. **Disadvantages:** Computationally expensive.
[11]	Towards LIDAR-RADAR Based Terrain Mapping	Guerrero et al.	2015	Explores the integration of radar and LiDAR for terrain mapping, providing enhanced navigation capabilities in unstructured outdoor environments. **Advantages:** Improved mapping in unstructured terrains. **Disadvantages:** Susceptible to sensor misalignment.
[10]	Learned Ultra-Wideband Radar Sensor Model for Augmented LiDAR-Based Traversability Mapping in Vegetated Environments	Ahtiainen et al.	2015	Ultra-wideband radar sensor models are used to augment LiDAR data, improving terrain mapping and object detection in vegetated environments. **Advantages:** Effective in dense vegetation. **Disadvantages:** Requires extensive model training.
[54]	Evaluation of a UWB radar interface for low power radar sensors	Genschow et al.	2015	The performance and integration of ultra-wideband (UWB) radar interfaces in low-power applications are examined. UWB radar technology is evaluated for its effectiveness in low power settings. **Advantages:** Low power consumption. **Disadvantages:** Limited range and resolution.

## Abbreviations

The following abbreviations are used in this manuscript:

ADAS	Advanced driver assistance systems
CI	Confidence intervals
CNN	Convolutional neural network
DNN	Deep neural network
EAS	Enterprise Estonia
GPU	Graphics processing unit
GPS	Global positioning system
IMU	Inertial measurement unit
LiDAR	Light detection and ranging
mmWave	Millimeter-wave
RUP	Programme for Applied Research
ResNet	Residual network
SAR	Synthetic-aperture radar
SLAM	Simultaneous localization and mapping
SRTM	Shuttle Radar Topography Mission
SVM	Support vector machine
UAV	Unmanned aerial vehicle
UGV	Unmanned ground vehicle
YOLO	You Only Look Once
